# Supplementary figures and images for: Prevalence and help-seeking for infertility in a population with a low fertility rate
Source: PLoS One. 2024 Jul 18;19(7):e0306572. doi: 10.1371/journal.pone.0306572 (PMC11257226; doi:10.1371/journal.pone.0306572)

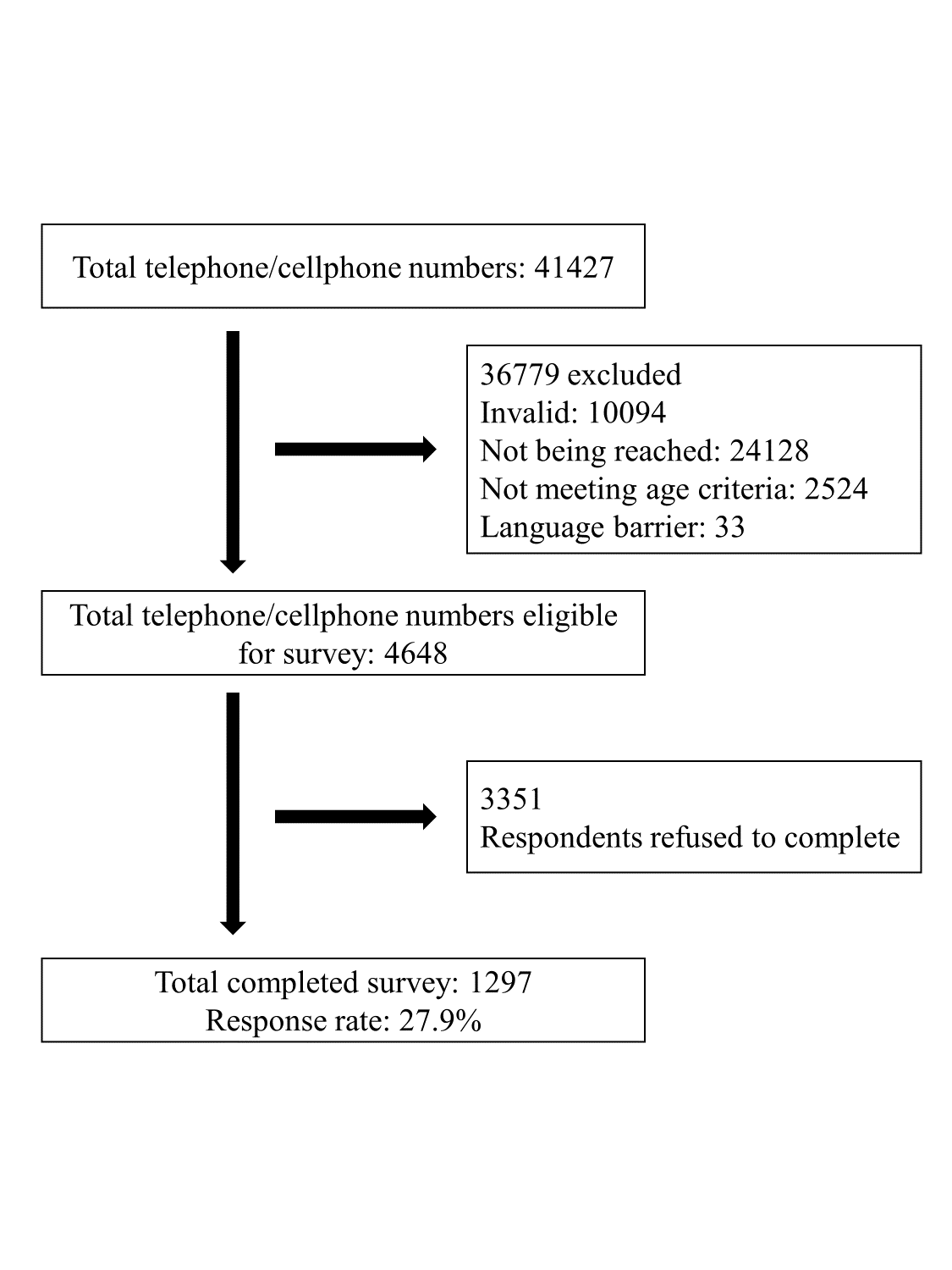


**Supplementary Figure S1.** Recruitment and participation of telephone survey

Supplement: S1 Fig — (DOCX) [file pone.0306572.s001.docx]
