# Supplementary material for: Prevalence and help-seeking for infertility in a population with a low fertility rate
Source: PLoS One. 2024 Jul 18;19(7):e0306572. doi: 10.1371/journal.pone.0306572 (PMC11257226; doi:10.1371/journal.pone.0306572)
Supplement: Attachment — Submitted filename: Review for Prevalence and Help seeking for infertility.docx [file pone.0306572.s002.docx]

Manuscript #: PONE-D-24-11968

Prevalence and Help-Seeking for Infertility in a Population with a Low Fertility Rate

Summary and Background:

The study was a cross sectional population-based study conducted through telephone interviews which sought to assess the prevalence of infertility as well as the number of people seeking help for fertility.

This was a commendable study as it sought to determine the current state of fertility and fertility

treatments in Taiwan giving their low total fertility rate per woman which is less than 1. Though

Taiwan was noted as being one of the countries facing a rapid decline in fertility, the study only

concentrated on determining the prevalence of primary infertility; secondary infertility was not

mentioned.

The background of the study was silent about the cultural and religious beliefs regarding fertility

in Taiwan as this would have given more insights the fertility seeking behaviour of the people.

Methodology:

Cross sectional studies are good in determining point prevalence and the number of people

reached were above 40,000 which is commendable. However, the response rate of 27.9% was

low and clear explanation for this was not given.

In the first and second definitions of infertility used in this study, it was not clear if the use of

contraception was the differentiating factor. Is it possible for ‘childless with no pregnancy

attempt’ to have it’s own place in the definitions of terms in this study; it appeared in both

groups?

Results and Discussion:

‘With regard to professional help-seeking, 11.6% of men and 10.7% of women had ever

consulted a doctor about getting pregnant; 10.4% of men and 9.5% of women had ever

received diagnostic tests/treatment to help with conceiving; 3.1% of men and 2.1% of women

were currently receiving diagnostic tests/treatment to help with conceiving’. In the above

statement does it mean that male infertility is high in Taiwan? How does this compare to other

Asian countries?

In the final recommendations in this study, the author wrote that:

‘These findings also suggest that there is a gap between those who are currently experiencing

infertility and those who are currently being treated; hence, we recommend raising awareness

of infertility and improving access to infertility healthcare.’ I think the recommendation should

go beyond this as there may be some other sociologic factors; be it cultural or based on the

norm, which is hindering people from having children. This calls for a wider survey to

get to the root cause why people who do not have children are reluctant to do so.

TO THE EDITOR: On a general note, this is a good study considering the paucity of studies from

Taiwan and it can be considered for publication if the above issues are addressed. Thank you.

PROF. HAJARATU UMAR SULAYMAN.

19^th^ May 2024.
